# A Simple Double-Spin Closed Method for Preparing Platelet-Rich Plasma

**DOI:** 10.7759/cureus.20899

**Published:** 2022-01-03

**Authors:** Edilson S Machado, Fabiano P Soares, Roberta S Yamaguchi, William K Felipone, Robert Meves, Tais Amara C Souza, Roberto Topolniak, José P Caldas, Ernani V Abreu, Luiz S Rabelo Neto, Pedro Vinicius S Pinchemel, Markus Bredemeier

**Affiliations:** 1 Pain Medical Center, Regenerar, Porto Alegre, BRA; 2 Faculty of Medicine, University of Porto, Porto, PRT; 3 Departamento de Ciências Fisiológicas, Faculdade de Ciências Médicas da Santa Casa de São Paulo, São Paulo, BRA; 4 Santa Casa Spine Center, Faculdade de Ciências Médicas da Santa Casa de São Paulo, São Paulo, BRA; 5 Public Health Institute, University of Porto, Porto, PRT; 6 Spine Group, Hospital Ernesto Dornelles, Porto Alegre, BRA; 7 Serviço de Reumatologia, Hospital Nossa Senhora da Conceição, Porto Alegre, BRA

**Keywords:** platelets, double-spin, growth factors, orthobiologics, orthopedics, regenerative medicine, platelet-rich plasma

## Abstract

Objective: To describe and analyze a new protocol for the extraction of platelet-rich plasma (PRP) for use in clinical practice and compare this technique with methods that have been previously described in the medical literature.

Methods: Sixteen blood samples from healthy volunteers were collected. PRP was prepared using our new double-spin technique, consisting of successive centrifugation of blood samples with two different spins, without opening the container. Descriptive analysis of cell counts in baseline and PRP samples was undertaken. Comparison between cell and platelet count in baseline and PRP samples, as well as the statistical analysis, were done.

Results: The mean platelet concentration ratio was 3.47 (SD: 0.85; 95% CI: 3.01-3.92; range: 2.48-5.71). The baseline whole blood platelet count correlated positively to the PRP platelet count (rP_ _= 0.56; 95% CI: 0.09-0.88; P = 0.023). The PRP was enriched for lymphocytes and monocytes but presented significantly lower counts of neutrophils and eosinophils in comparison to baseline.

Conclusion: Results show a safe and easily reproducible method to obtain PRP for use in clinical daily practice.

## Introduction

The application of platelet-rich plasma (PRP) has emerged as a safe and viable alternative to surgical procedures in orthopedics. PRP is defined as a volume of autologous plasma that has a platelet concentration above baseline [1]. The term PRP was created in the 1970s by hematologists and was initially used to treat patients with thrombocytopenia. PRP is currently used in different medical fields [2], like maxillofacial surgery, orthopedics, and dermatology. There are now several high-quality studies about the use of PRP in these specialties, specifically in the field of treatments for musculoskeletal diseases. In the last decades, PRP has gained increased attention in orthopedic and sports medicine [3-5] and has been advocated by several researchers as a potentially effective therapy to control degenerative disease in joints like the knee, spine, and hip [6-8], and has been subject to great interest in the current research agenda [9]. The therapeutic effects of PRP are attributed to the high concentration of growth factors present in platelets, like vascular endothelial growth factor (VEGF), platelet-derived growth factor AB (PDGF-AB), and transforming growth factor beta 1 (TGF-β1), which are responsible for providing regenerative stimulus that promotes tissue repair [10] by cell proliferation, angiogenesis, and cell migration [11].

Previous studies have reported different methods of obtaining PRP using different centrifugation speeds and varying numbers of spins [12]. Currently, there are several commercial devices available for the preparation of PRP [13] and there are dozens of descriptions of single or double-spin handmade techniques. Some investigators have proposed standard protocols for evaluating the quality of PRP, mainly for reproducibility purposes and for the inclusion of studies in systematic reviews and meta-analyses. In general, PRP preparation in clinical studies involves complex and expensive protocols, and most studies do not provide sufficient information to allow for adequate reproducibility. Therefore, a detailed, precise, and stepwise description of the PRP preparation protocol is required to allow comparisons between studies [12].

The lack of standardization and the high cost of preparing the PRP using the standard commercial kits led our research team to look for a simpler, safer, and cheaper technique. After reviewing and testing the main published protocols, we developed a closed system, with a double centrifugation technique, obtaining a satisfactory platelet concentration [14].

Therefore, in the present study, we aimed to describe a simple, safe, inexpensive, and reproducible protocol for obtaining PRP for use in research and clinical practice based on modifications of the technique previously described by our group.

## Materials and methods

Healthy volunteers were invited to participate in the study, and after an explanation of the study purpose and obtaining written informed consent, their blood was collected from the antecubital vein. Blood was collected first in an ethylenediaminetetraacetic acid (EDTA) containing tube (BD Vacutainer EDTA, Ref. 367861, BD, Franklin Lakes, NJ) for baseline counts of platelets, red blood cells, and leukocytes in each patient. For PRP preparation, blood was collected using a collection tube (BD Vacutainer ACD Solution A, Ref. 364606) containing 3.2% sodium citrate at a volumetric ratio of 1.5 ml of anticoagulant to 8.5 ml of blood. Immediately after the blood collection, these tubes were placed for centrifuging. A serologic centrifuge (Kasvi model K14-0815A, Kasvi, Paraná, Brazil) with a 36° fixed angle rotor was used (rotor radius: maximum 92 mm). With the aim of creating a protocol for everyday clinical use, the experiments that we did were conducted at room temperature (22°C), and the centrifuges were not refrigerated.

**Figure 1:**
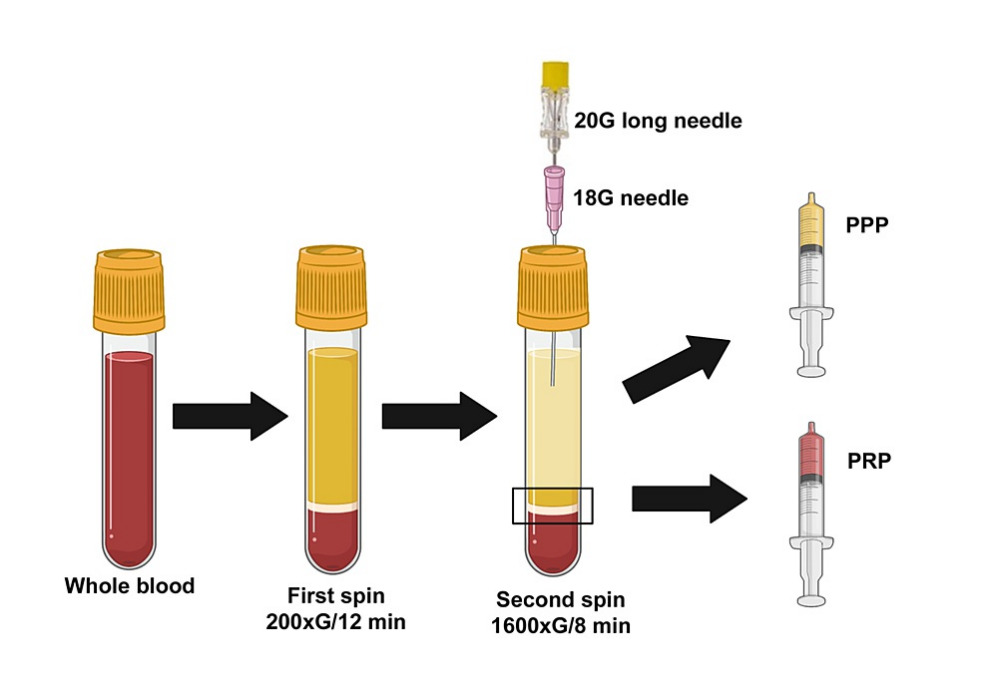
Proposed centrifugation method. After whole blood is collected from volunteers, it undergoes the first spin at 200G for 12 minutes. Then, without any manipulation, it goes through the second spin at 1600G for eight minutes. Finally, with the assistance of an 18G needle (used as a guide), a 20G long needle is inserted to aspirate the poor platelet plasma and the platelet-rich plasma. Created with BioRender.com. PPP, platelet-poor plasma; PRP, platelet-rich plasma.

The centrifugation protocol is as follows (Figure 1): (1) first spin of 200G for 12 minutes; (2) no manipulation of the tubes and neither plasma extraction is done after this spin and the tubes are just kept in the centrifuge; (3) second spin of 1600G for eight minutes; (4) after the second spin, gently remove the tube from the centrifuge, without tilting or shaking; (5) put the tube in support; (6) perform asepsis of the tube lid with alcohol or chlorhexidine-alcohol; (7) insert an 18G needle that will serve to guide a longer needle (the outer needle will allow the entry of the inner needle more smoothly, without friction); (8) with a 20G long needle (70 mm), aspirate approximately 3.5 ml of the upper plasma layer (platelet-poor plasma); (9) finally, aspirate the approximately 1 ml of remaining plasma, including the buffy coat, just above the hematic layer. PRP is now ready.

To reproduce daily clinical practice, the analysis of the concentration of blood components was performed immediately after the preparation. The whole blood and PRP samples were analyzed on a Sysmex XE5000 hematology counter (Sysmex, Hyogo, Japan). The platelet concentration factor was calculated as the ratio between platelet counts in PRP and whole blood.

Analysis of data was done using IBM SPSS Statistics for Windows, version 20.0 (IBM Corporation, Armonk, NY). Continuous variables were presented as mean and standard deviation (SD). Variables representing cell counts were tested for normality of distribution using the Kolmogorov-Smirnov test, and these variables did not deviate significantly from a normal distribution (P > 0.20 for all variables). Cell counts at baseline and in PRP were compared using Student’s t-test for paired samples. Correlation between the baseline and the PRP platelet counts was calculated using Pearson’s r coefficient (rP), and the 95% confidence interval was estimated using bootstrapping. Two-tailed P-values equal to or less than 0.05 were considered statistically significant.

The present study was approved by the institutional review board of our institution (Hospital Nossa Senhora da Conceição, Grupo Hospitalar Conceição) and registered at Plataforma Brasil (the Brazilian government’s registry of scientific studies) under CAAE (‘Certificado de Apresentação de Apreciação Ética’, Certificate of Presentation of Ethic Appreciation) number 53166416.1.0000.5530.

## Results

Sixteen healthy blood donors aged 19 to 53 years (mean: 39.9 years, SD: 9.9 years; nine females and seven males) volunteered to participate. The baseline counts of the blood components were within the normal range of values. A comparison between baseline and PRP cell counts is shown in Table 1. The mean platelet concentration ratio was 3.47 (SD: 0.85; 95% CI: 3.01-3.92; range: 2.48-5.71). The baseline whole blood platelet count correlated positively to the PRP platelet count (rP = 0.56; 95% CI: 0.09-0.88; P = 0.023; see Figure 1). The PRP was enriched for lymphocytes and monocytes but presented significantly lower counts of neutrophils and eosinophils in comparison to baseline (Table 1).

**Table 1:** Cell counts at baseline and after PRP preparation. * Counts are expressed as number x 103/μL, except when indicated otherwise. ** Paired Student’s t-test. PRP: platelet-rich plasma.

	Baseline values		Platelet-rich plasma		P-value**
	Mean ± SD	Range	Mean ± SD	Range	
Platelets	251.25 ± 62.29	158‒348	847.25 ± 206.20	553‒1216	<0.001
Red blood cells (x 10^6^/μL)	4.62 ± 0.57	3.46‒5.50	0.62 ± 0.64	0.07‒2.05	<0.001
White blood cells	7.62 ± 1.66	5.26‒9.68	10.40 ± 6.10	2.92‒26.09	0.137
Neutrophils	4.05 ± 1.04	2.44‒5.78	1.60 ± 1.30	0.43‒4.37	<0.001
Lymphocytes	2.78 ± 0.93	1.47‒4.37	7.12 ± 3.96	1.69‒18.16	0.001
Eosinophils	0.17 ± 0.10	0.06‒0.38	0.03 ± 0.03	0.00‒0.11	<0.001
Basophils	0.03 ± 0.01	0.02‒0.05	0.07 ± 0.09	0.00‒0.29	0.091
Monocytes	0.58 ± 0.21	0.35‒0.99	1.56 ± 1.21	0.43‒4.79	0.006

## Discussion

Normally, PRP is processed through a double-spin technique. First, there is the “soft” spin (low speed), which separates red cells from plasma, resulting in three layers: the supernatant acellular plasma, the buffy coat with the platelet concentrate, and the red cell concentrate underneath [15]. Then, the “hard” spin (high speed) is performed, separating platelets and white blood cells from plasma and thus isolating the PRP from the poor-platelet plasma (PPP) [1].

In this article, we describe and test a double-spin, closed system technique for obtaining PRP, using standard laboratory material, without a commercial kit. Our method differs from others previously reported by the absence of any kind of manipulation between the two cycles of centrifugation.

PRP can be obtained using a commercial kit or using a handmade technique. Because of the high variability in the production techniques and concentration/composition results [16], some PRP classification methods have been proposed. Delong et al. [17] postulated that an ideal PRP classification would be based on the absolute number of platelets, activation method, and leukocytes counts (PAW). A few years later, Mautner et al. [18] described a more refined and detailed classification system (platelet count, leukocyte presence, red blood cell presence, and use of activation [PLRA]), where the authors demonstrated that it is important to describe the absolute platelet count, leukocyte content, the percentage of neutrophils, presence of red blood cells, and whether exogenous activation was used. Magalon et al. [19] published the DEPA (Dose of injected platelets, Efficiency of production, Purity of the PRP, Activation of the PRP) classification based on the dose of injected platelets, the purity of the PRP obtained, and the activation process utilized. Lana et al. [20] introduced the MARSPILL classification system, with a significant focus on peripheral blood mononuclear cells. This classification describes the method (custom-made or commercial kit), activation, presence of red and white blood cells, number of spins, platelet concentration and number, light activation, and image guidance of the application. ﻿It focuses on mononuclear cells too, suggesting their action on neovasculogenesis and cellular proliferation. The advantage of this latter classification rests in the most comprehensive analysis of components and factors that can possibly influence the regenerative process, besides the platelet count.

The exponential growth of the number of studies on PRP therapy is a consequence of its widespread use in various medical fields. However, the diversity of protocols for PRP preparation causes difficulties in comparing and/or reproducing results. Chahla et al. [12] conducted a systematic review of studies investigating the use of PRP for the treatment of orthopedic pathologies and indicated that only 10% of these studies provided comprehensive reporting that included a clear description of the preparation protocol. They concluded ﻿that a detailed, precise, and stepwise description of the PRP preparation protocol is required to allow comparisons between studies and enable reproducibility.

An experts’ consensus was published listing the minimum data that should be reported in studies to favor standardization of the analyses [21]. The Minimum Information for Studies Evaluating Biologics in Orthopaedics (MIBO) statement is available at the following web address: www.mibo-statement.org. More recently, an association of orthopedic scientific societies was created to foster coordinated efforts, advocating the responsible use of biologics [9]. Nevertheless, despite numerous calls for minimal reporting standards in clinical studies regarding PRP preparation, only a third of the studies have provided these details [22].

Regarding the centrifugation process, there is a difference in the separation of the blood components in each spin of the double spin technique. The first spin (or soft spin), with low centrifugal force, is used to separate three layers from whole blood: red blood cells, buffy coat, and plasma. The second spin (or hard spin), with higher centrifugal force, is used to concentrate the platelets at the bottom part of the plasmatic phase [23].

In a review of different PRP protocols, Harrison et al. [24] found that there is a difference in the white blood cells’ concentration with one and two spin preparations. They found a depletion of granulocytes in single spin methods. In our technique, we found an increase in lymphocyte and monocyte concentration (Table 1). Regarding the mass of each cell type that compounds the whole blood, lymphocytes and monocytes are closer to platelets than other components.

In our technique, after the first low-speed centrifugation, the sample remains in the original tube (closed system), avoiding excessive manipulation and mitigating contamination risk. The second centrifugation is performed at a higher speed, just after the end of the first one. In the present study, we upgraded the turn down-turn up technique described in Machado et al. [14], facilitating its reproducibility and reducing preparation time. This two-spin method follows the principles of low-speed centrifugation to preserve the maximum volume of platelets, leukocytes, and growth factors and uses discontinuous centrifugation to modulate and control platelet recovery [25]. Our technique has the advantages of a closed system but with less cost and higher biological security, with a low risk of contamination. Considering the current legislation present in several countries, which requires a complex laboratory structure when using an open system for the preparation of PRP, this technique brings a clear advantage as it is fully performed in a closed system, reducing the risk of contamination. The simplicity of the technique also makes it easily reproducible, facilitating the standardization of lab procedures in multicentric studies.

The present study also has limitations. We obtained a mean platelet concentration ratio of 3.47, varying from 2.48 to 5.71. So, despite using blood exclusively from healthy volunteers, there was a substantial variability of concentrating ability of the technique between individuals. However, compared to other described techniques and current guidelines, our figures are within an acceptable range of values [2]. We did not measure the final concentrations of growth factors in our PRP preparations, which are thought to be important factors in the therapeutic effects of orthobiologics. The procedures of PRP extraction in this study were all performed by the same investigator (ESM) who developed the method, and so our results must be confirmed by other groups of researchers before being considered definitive. We also did not test our product in a real clinical setting, and so further studies on this technique are still needed.

## Conclusions

To date, this is the first publication that presents a double-spin, closed system using standard laboratory material, without a commercial kit, for the preparation of PRP. Our method is simple, safe, and uses exclusively low-cost devices. Our results were consistent with current standards of quality for PRP preparation for clinical trials suggested by the American Academy of Orthopaedic Surgeons working group. Compared with other currently employed techniques, our method may have significant advantages. The proposed technique is not time-consuming, does not require highly trained personnel, does not demand expensive laboratory kits or a refrigerated centrifuge, and has a low risk of contamination considering the use of a closed system.
